# Cheilitis Glandularis: A Report of Two Cases

**DOI:** 10.7759/cureus.116290

**Published:** 2026-09-15

**Authors:** Ramachandra Reddy Gowda Venkatesha, Karthik Rajaram Mohan, Vasu Sridharrao, Sindhuja Rajalingam, Saramma Mathew Fenn

**Affiliations:** 1 Oral Medicine and Radiology, Vinayaka Mission's Sankarachariyar Dental College, Vinayaka Mission's Research Foundation (Deemed to be University), Salem, IND; 2 Oral Medicine and Radiology, Vinayaka Mission's Sankarachariyar Dental College, Vinayaka Mission's Research Foundation (DU), Salem, IND

**Keywords:** adoloscent girl, cheilitis glandularis, tacrolimus, vermilion border of lip, vermilionectomy

## Abstract

Cheilitis glandularis is a rare benign disorder affecting the vermilion border of the lower lip, the occurrence of which is unknown and still remains a myth. some attributed its formation because of the increased expression of surface aquaproteins, hyperplasia from chronic irritation by lip biting, and ultraviolet rays from the sun resulting in squamous epithelial metaplasia but unrelated to inflammation. This article describes the classical clinical picture of cheilitis glandularis in two cases, one related to amelogenesis imperfecta, which is significant for understanding the diagnosis and improving overall patient's health.

## Introduction

Cheilitis glandularis is a rare benign disorder affecting the vermilion border of the lower lip that occurs as a result of an unknown etiology unrelated to inflammation of minor salivary glands [1]. Cheilitis glandularis is often misdiagnosed, as it can be confused with other lip swellings caused by actinic cheilitis, self-inflicted injuries such as Munchausen syndrome, or lip swellings caused by food or drug allergies such as angioedema [2]. The etiology of cheilitis glandularis remains unknown and controversial. Cheilitis exfoliativa is characterized by repeated desquamation of dry scales of keratin on surface of lip, which on removing leaves a raw area of erythema [3,4]. Cheilitis granulomatosa is characterised by recurrent lip swellings along with fissured tongue and facial paralysis as in Melkerson-Rosenthal syndrome [5]. The case report describes the classical clinical picture of cheilitis glandularis in two cases, one related to amelogenesis imperfecta, which is significant for understanding the diagnosis and improve overall patient's health.

## Case presentation

Case one

A 14-year-old female reported for a routine dental checkup. History revealed no prodromal symptoms of fever. Extraoral clinical examination revealed diffuse swelling resembling the appearance of a dumbbell of size 6cm x 2 cm with focal areas of redness (Figure 1).

**Figure 1:**
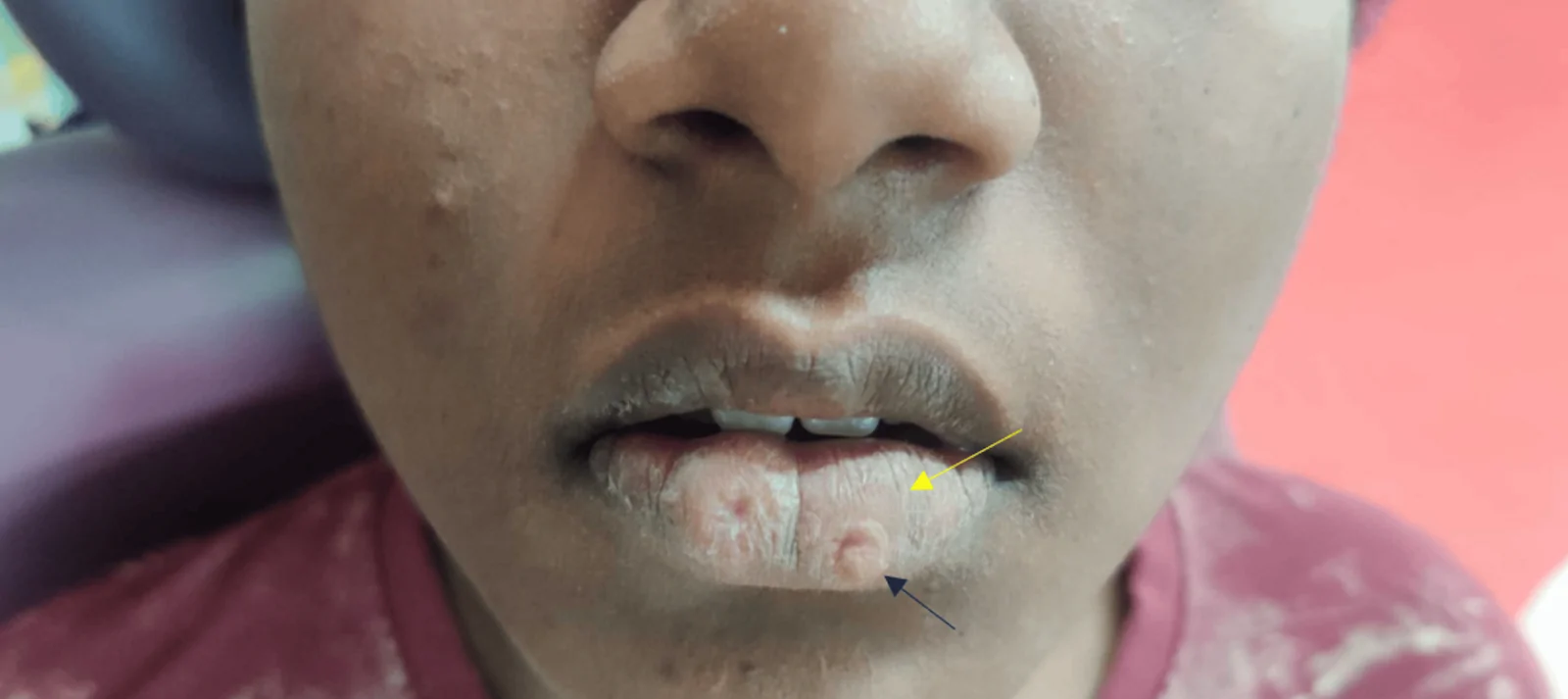
Case 1: Extraoral examination revealed a dumb-bell shaped swelling involving vermilion border of lip with surface dryness and discrete focal dilated ostia of minor salivary glands on the vermilion border of lower lip

On palpation, the swelling involving vermilion border of lower lip was non-tender, soft in consistency. The swelling returns to its shape after palpation (Video 1). Based on the above clinical findings, a provisional diagnosis of cheilitis exfoliativa was made.

**Video 1:** Cheilitis glandularis palpation

Case two

An 18-year-old teen boy reported for a routine dental checkup. Extraoral clinical examination revealed macrochelia of size 6 cm x 2.5cm involving the vermilion border of the lower lip (Figure 2).

**Figure 2:**
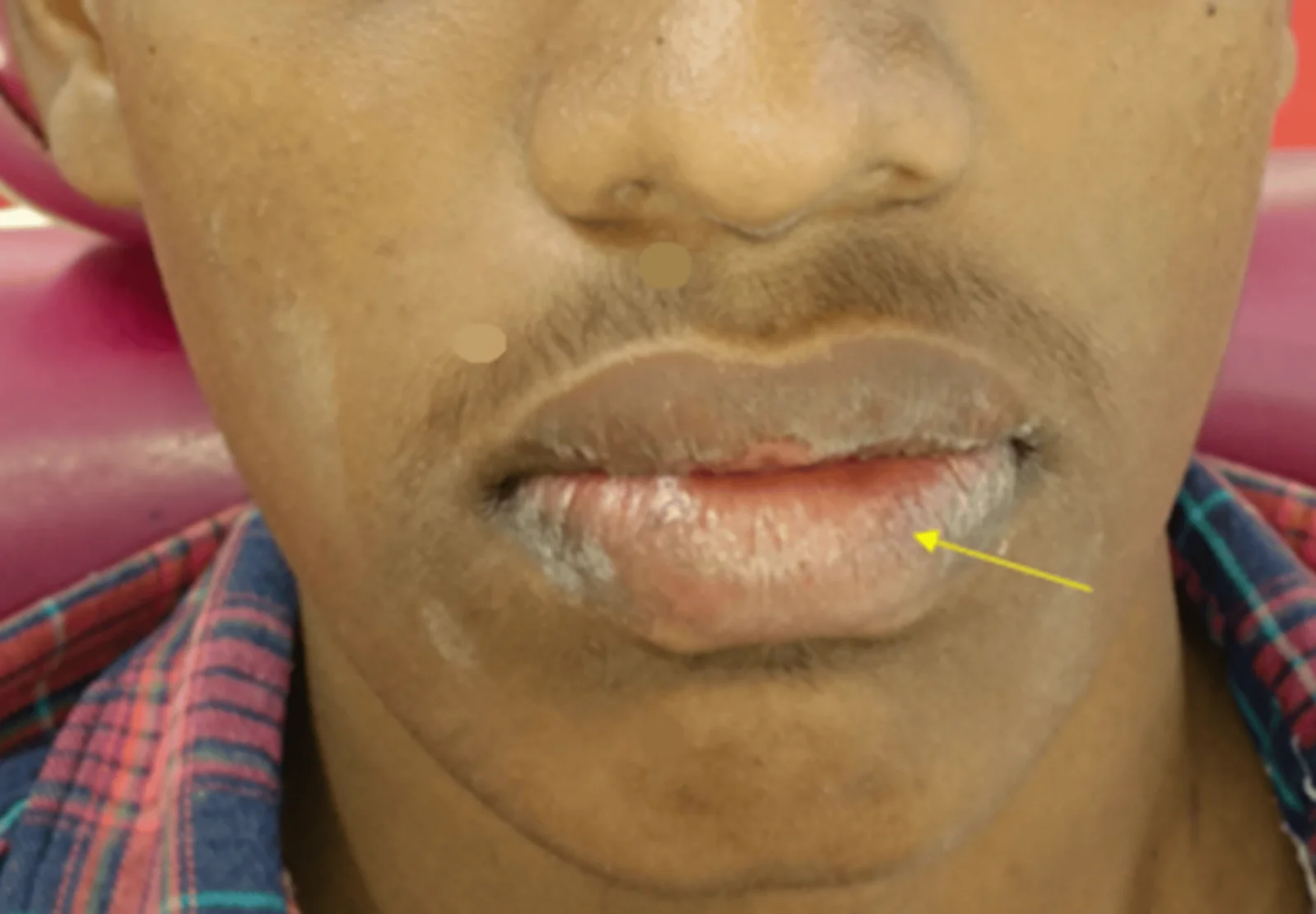
Case 2: Extraoral examination revealed diffuse swelling of the vermilion border of lower lip with surface excoriations

History revealed it was present from birth but became more prominent during his pubertal age. Extraoral examination revealed a diffuse swelling involving vermilion border of the lower lip. Intraoral examination revealed malformed stained maxillary and mandibular anterior teeth with open contact points (Figures 3A, 3B).

**Figure 3:**
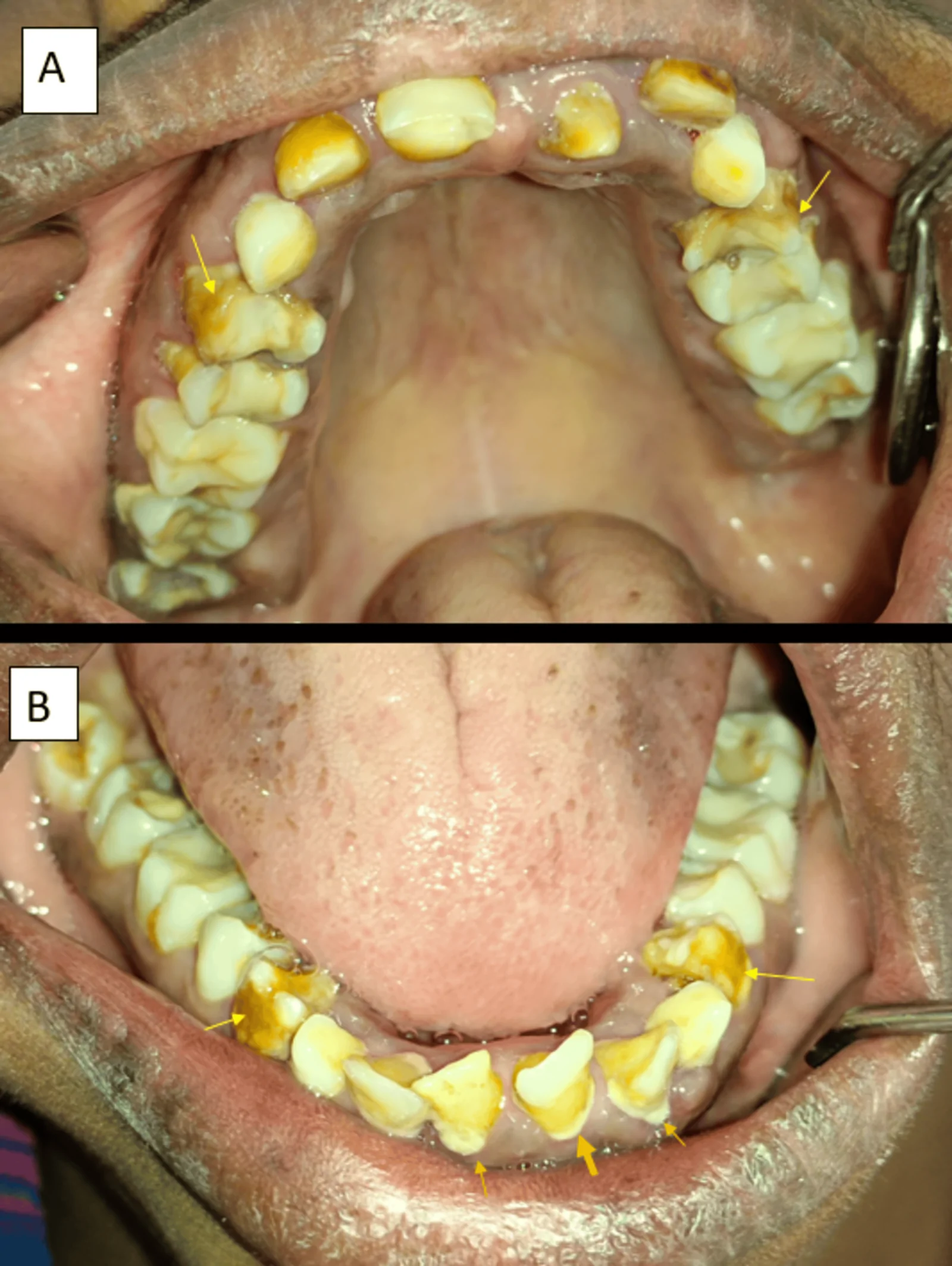
Case 2: (A) Intraoral examination revealed open contact points with loss of enamel on the incisal aspects of maxillary anterior teeth. (B) Intact enamel only in the cervical margin of mandibular incisor and premolar teeth.

Orthopantomography revealed the presence of impacted canine in both maxilla and mandible (Figure 4).

**Figure 4:**
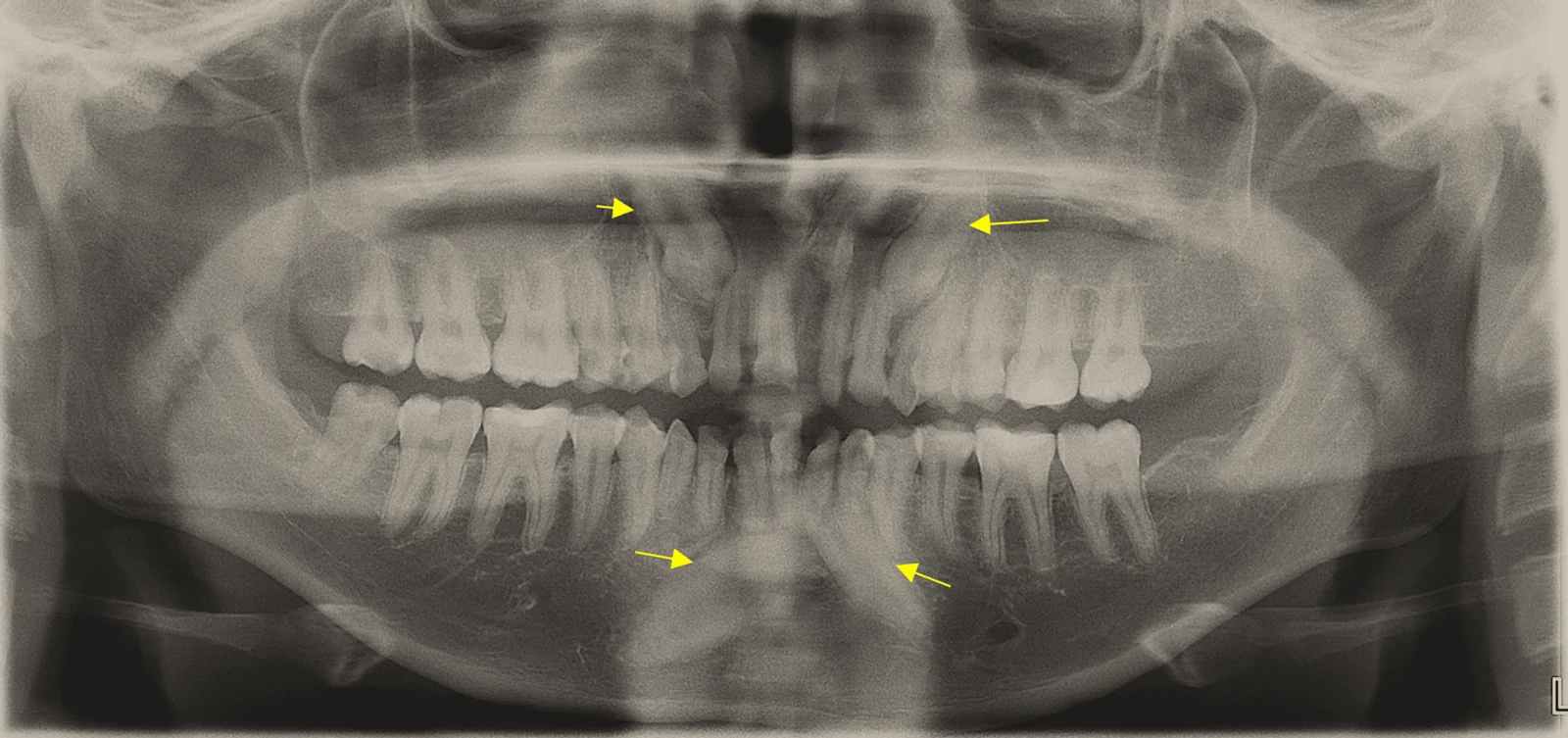
Case 2: Orthopantamography revealed presence of impacted canine in both maxilla and mandible and open contact points between his teeth

The differential diagnosis for both of the above-mentioned cases includes self-inflicted injuries (Munchausen syndrome), actinic cheilitis, and angioedema caused by food or drug allergies. The uniqueness of the case report is that cheilitis glandularis was reported in a 14-year-old adolescent girl and an 18-year-old adolescent boy in occurrence with multiple impacted teeth, amelogenesis imperfecta. The limitations of this case report are that, since the lesion is painless, the patient did not give consent either for topical tacrolimus application or vermilionectomy procedure, and treatment follow-up was not done. Further radiological investigation such as the position of the impacted canine not analyzed by three-dimensional radiographic visualization by cone beam computed tomography. No histopathological findings or special immunohistopathological sections were provided in this case report. 

## Discussion

Etiology

The variable and increased expression of surface aquaproteins, specifically Aquaporins 1, 2, and 8, is attributed to the formation of cheilitis glandularis [1]. Cheilitis glandularis is a rare benign disorder affecting the vermilion border of the lip with an unknown cause of inflammation [2]. Cheilitis glandularis is characterized by macrocheilia of variable severity and the presence of discrete red areas that represent dilated openings of minor salivary glands of the vermilion border on the lower lip [2]. The disease was first described by von Volkmann under other synonyms such as “cheilitis glandularis apostematosa” or “myxadenitis labialis.” The appearance, depth, and size of minor salivary glands in patients with cheilitis glandularis were not different from those in control subjects studied by Swerlick and Cooper [3].

Clinical types

The three different types of cheilitis glandularis include the simple, superficial suppurative type (Bazel's disease) and the deep suppurative type (Glandularis apostematosa). This disease can manifest in many different ways, ranging from asymptomatic swelling of the lip to focal discrete areas of redness or surface exfoliations [3]. In this condition, thick saliva or mucopurulent secretions exit through dilated ducts along the vermilion border, accompanied by varying levels of macrocheilia [4].

Histopathological findings

A proliferative pattern of squamous hyperplasia with papillary architecture, and adenomatous hyperplasia with enlarged ducts is a predominant histological finding in cheilitis glandularis [6-8].

Diagnostic applications

The diagnostic application of in vivo confocal reflectance microscopy in one reported case of cheilitis glandularis was poor due to the small number of descriptive cases [9]. The angioedema of the lip is confirmed by a positive patch test. A history of exposure to sunlight reveals actinic cheilitis. History of repetitive lip biting and occurrence of mucosal bleeding, cicatrization of lip reveals Munchausen syndrome of proxy. History of exposure to drugs reveals drug-induced cheilitis.

Differential diagnosis

Differential diagnosis for cheilitis glandularis is given in Table 1.

**Table 1:** Differential diagnosis for cheilitis glandularis

Differential diagnosis	Clinical features
Angioedema	Non-pitting, asymmetrical diffuse enlargement of the lip. It can involve other parts of the body such as the face and extremities.
"artifactual cheilitis" “Munchausen syndrome of proxy” or factitious cheilitis"	Repetitive lip biting, mucosal bleeding, ulcerations on the buccal mucosa, cicatrization of the affected lip, tooth loss
cheilitis exfoliativa" or "tic de levres"	Persistent flaking and scaling due to desquamation of superficial keratin. When these scales are removed a normal layer is revealed with associated pain, erythema, and edema.
Plasma cell cheilitis	The lips appear dry and fissured with dark red plaque or atrophic areas.
Cheilitis granulomatosis	recurrent firm swelling of one or both lips
Drug-induced cheilitis	Common causes of drug-related cheilitis include retinoids such as Etretinate, topical synthetic retinoids Isotretinoin, Protease inhibitors such as indinavir

Treatment modalities

Topical application or intralesional injection of 0.1% w/w Tacrolimus (Tacroz forte, Glenmark Pharmaceuticals Ltd., Mumbai, India) or vermilionectomy is the preferred surgical treatment modality that removes damaged vermilion and reduces macrocheilia [10]. Volonte reported a case of suppurative cheilitis glandularis in a 13-year-old pediatric age group treated with doxycline and clarithromycin antibiotic therapy [11]. Metal ceramic crowns aid in restoring the lost vertical dimension of posterior teeth, and zirconia or all-ceramic crowns are advised for anterior teeth affected by amelogenesis imperfecta [12]. The various non-surgical (pharmaceutical therapies) and surgical therapy for cheilitis glandularis are discussed (Table 2).

**Table 2:** Treatment modalities for cheilitis glandularis

Non-surgical ( Pharmaceutical therapies)	
Topical	Tacroz, Glenmark Pharmaceuticals Limited (0.1% tacrolimus ointment) twice daily for two weeks
	ELIDEL, Novartis Pharmaceuticals. Pimecrolimus ( 1% cream) twice daily for two weeks
	Combined oral minocycline (100 mg once per day) plus tacrolimus ointment 0.1% twice daily for 6 weeks.
Intralesional	Intralesional injections of 10 mg triamcinolone suspension in one-month interval
Systemic	Oral Prednisone at 40 mg/day for 10 days Doxycycline 100 mg once daily / Clarithromycin 500 mg once daily for 7 days
Surgical	Vermilionectomy (Lip shave)

## Conclusions

The diagnosis of cheilitis glandularis remains challenging to the dentist as it can manifest clinically in different forms such as painless swelling to surface excoriations with focal discrete red areas that represent dilated ostia of minor salivary glands on the vermilion border of the lip. Cheilitis glandularis may be misdiagnosed as other forms of lip swellings such as cheilitis granulomatosa, cheilitis exfoliativa, drug-induced cheilitis, periapical odontogenic infections from carious mandibular teeth, sialadenitis of minor salivary glands, angioedema, self-inflicted injuries such as Munchausen syndrome of proxy and actinic cheilitis with which it coexists and can lead to a diagnostic challenge to the clinician. Dentists must be aware of this condition to improve overall health.

## References

[1] Nico MM, Melo JN, Lourenço SV (2014). Cheilitis glandularis: immunohistochemical expression of protein water channels (aquaporins) in minor labial salivary glands. J Eur Acad Dermatol Venereol.

[2] Oliver ID, Pickett AB (1980). Cheilitis glandularis. Oral Surg Oral Med Oral Pathol.

[3] Nico MM, Nakano de Melo J, Lourenço SV (2010). Cheilitis glandularis: a clinicopathological study in 22 patients. J Am Acad Dermatol.

[4] de Sales SC, Oliveira SR, Pantuzzo ES (2023). Cheilitis glandularis: case report and systematic literature review. Int J Dermatol.

[5] Wehl G, Rauchenzauner M (2018). A systematic review of the literature of the three related disease entities cheilitis granulomatosa, orofacial granulomatosis and Melkersson-Rosenthal syndrome. Curr Pediatr Rev.

[6] de Morais Medeiros HC, Del Carmen Martinez Vargas Y, Gonzaga AK, Queiroz LM, de Medeiros AM, de Oliveira PT, da Silveira ÉJD (2021). Co-existence of cheilitis glandularis and actinic cheilitis-an update. Oral Maxillofac Surg.

[7] Stoopler ET, Carrasco L, Stanton DC, Pringle G, Sollecito TP (2003). Cheilitis glandularis: an unusual histopathologic presentation. Oral Surg Oral Med Oral Pathol Oral Radiol Endod.

[8] Reichart PA, Scheifele Ch, Philipsen HP (2002). Glandular cheilitis. 2 case reports (Article in German). Mund Kiefer Gesichtschir.

[9] Maher NG, Collgros H, Uribe P, Ch'ng S, Rajadhyaksha M, Guitera P (2016). In vivo confocal microscopy for the oral cavity: current state of the field and future potential. Oral Oncol.

[10] Sugaya N, Migliari D (2018). Cheilitis glandularis of both lips: successful treatment with a combination of an intralesional steroid injection and tacrolimus ointment. Case Rep Dent.

[11] Volonté M, Barbaccia V, Bonelli A, Brazzelli V (2023). Deep suppurative glandular cheilitis: a pediatric case report. JAAD Case Rep.

[12] Roma M, Hegde P, Durga Nandhini M, Hegde S (2021). Management guidelines for amelogenesis imperfecta: a case report and review of the literature. J Med Case Rep.

